# Identification of quantitative trait loci for tillering, root, and shoot biomass at the maximum tillering stage in rice

**DOI:** 10.1038/s41598-022-17109-y

**Published:** 2022-08-03

**Authors:** Jinyoung Y. Barnaby, Anna M. McClung, Jeremy D. Edwards, Shannon R. M. Pinson

**Affiliations:** USDA Agricultural Research Service, Dale Bumpers National Rice Research Center, Stuttgart, AR 72160 USA

**Keywords:** Genetics, Plant sciences

## Abstract

Tillering and plant biomass are key determinants of rice crop productivity. Tillering at the vegetative stage is associated with weed competition, nutrient uptake, and methane emissions. However, little information is available on quantitative trait loci (QTLs) associated with tiller number (*qTN*), root biomass (*qRB*), and shoot biomass (*qSB*) at the active tillering stage which occurs approximately 6 weeks after planting. Here, we mapped tiller and biomass QTLs with ~ 250 recombinant inbred lines derived from a ‘Francis’ by ‘Rondo’ cross using data collected at the maximum tillering stage from two years of greenhouse study, and further compared these QTLs with those mapped at the harvest stage from a field study. Across these three studies, we discovered six *qTNs*, two *qRBs*, and three *qSBs*. Multiple linear regression further indicated that *qTN1-2*, *qTN3-3*, *qTN4-1*, *qRB3-1*, and *qRB5-1* were significant at the maximum tillering stage while *qTN3-2* was detected only at the harvest stage. Moreover, *qTN3-1* was consistently significant across different developmental stages and growing environments. The genes identified from the peak target *qTN* regions included a carotenoid metabolism enzyme, a MYB transcription factor, a CBS domain-containing protein, a SAC3/GANP family protein, a TIFY motif containing protein, and an ABC transporter protein. Two genes in the qRB peak target regions included an expressed protein and a WRKY gene. This knowledge of the QTLs, associated markers, candidate genes, and germplasm resources with high TN, RB and SB is of value to rice cultivar improvement programs.

## Introduction

Over 80% of the U.S. rice acreage is located in the Mid-south with the remainder in California^1^. Cultivars grown in these two regions are derived from the Japonica sub-species; primarily, tropical *japonica*, long grain cultivars in the Mid-south and temperate *japonica-*derived medium grain cultivars in the West^2^. U.S. rice breeding efforts have focused on these two genetic sub-populations with very limited utilization of germplasm from other gene pools^3^. However, because the *indica* sub-population has been demonstrated to have greater allelic diversity, it has been proposed as a means of improving tropical and temperate *japonica* based varieties^4–7^.

Tillering is a key determinant of grain yield and biomass in rice and tillering at an early vegetative stage is important for weed competition^8^. Progress has been made in identifying quantitative trait loci (QTLs) and genes important in production of tillers in rice that can be used in varietal improvement programs^9–18^. However, although QTLs have been widely identified, studies have shown that tiller development is regulated by a complex network driven by genetic and hormonal factors (e.g., auxins, cytokinins, abscisic acid, gibberelins, and strigolactones) that are sensitive to environmental fluctuations^19–23^. Therefore, identifying QTLs that are robust across different genetic backgrounds and environments is of importance.

In general, *japonica* rice cultivars have fewer tillers compared to *indica* cultivars^9,10,24–26^. Studies using *indica* x *japonica* mapping populations have demonstrated high yield attributed to increases in tiller number (TN) coming from the *indica* parent^10,18,24,25,27,28^. Previous studies indicated that several TN-QTL were more strongly detected at the 5- to 6-week-old seedling stage than at or after heading^18,28–30^, and further study showed that selection for increased tillering at 5- to 6-week-old vegetative stage resulted in increases in tillering at maturity, panicle number, and grain yield under drill-seeded field conditions^11^. Recently, Barnaby et al.^31^, using a small set of microsatellite markers, demonstrated that among high and low tillering recombinant inbred lines (RILs) from three *indica* × *japonica* mapping populations, 12 tillering QTLs were identified and the *indica* allele was associated with high tillering in all but one QTL of one population.

Another important agronomic trait is the root system which is responsible for water and mineral uptake, competition with weeds, and tolerance to some biological pests. There have been a few studies that have identified QTLs affecting root structure and development in rice^32–37^ largely due to the difficulty of analyzing belowground plant traits and the variable influence of the growing environment. Kim et al.^38^ demonstrated that rice varieties with increased root area have high methane emissions. Rice root and shoot biomass have been shown to influence soil microbiome structure influencing nutrient uptake, plant health, and methane emissions^39^. Thus, exploring the interrelationship of tillering, shoot biomass and root biomass is integral to understanding productivity in rice as well as the interaction of the rice plant with the environment.

‘Rondo’ (PI657830)^40^ is a long-grain *indica* cultivar having high tillering and high yield potential, excellent disease resistance, superior parboiling grain quality, and weed suppression potential^26^. As a means of mapping and introgressing *indica* QTLs for these and other traits, a mapping population of 250 RILs was developed by crossing Rondo with the low-tillering tropical *japonica* U.S. cultivar, Francis (PI632447)^41^, and a 7K-Rice SNP Array (C7AIR) was used to genotype the Francis X Rondo RIL population (FR-RILs) in the F_10_ generation as detailed in the Methods section. The aim of the present study was to investigate the genetics underlying the high yield potential in Rondo as related to TN, RB, and shoot biomass (SB). Three specific goals were to (i) identify QTLs associated with production of TN, RB, and SB at the 6-week-old seedling stage, i.e. maximum tillering (MT) stage, in greenhouse studies; (ii) evaluate the robustness of the QTLs identified at MT stage by comparing with QTLs identified at harvest (H) stage in a field study; and (iii) identify candidate genes located in the target peak areas of TN-, RB- and SB-QTLs that were identified.

## Results and discussion

### Comparison of TN, RB, and SB across greenhouse and field studies

**Figure 1:**
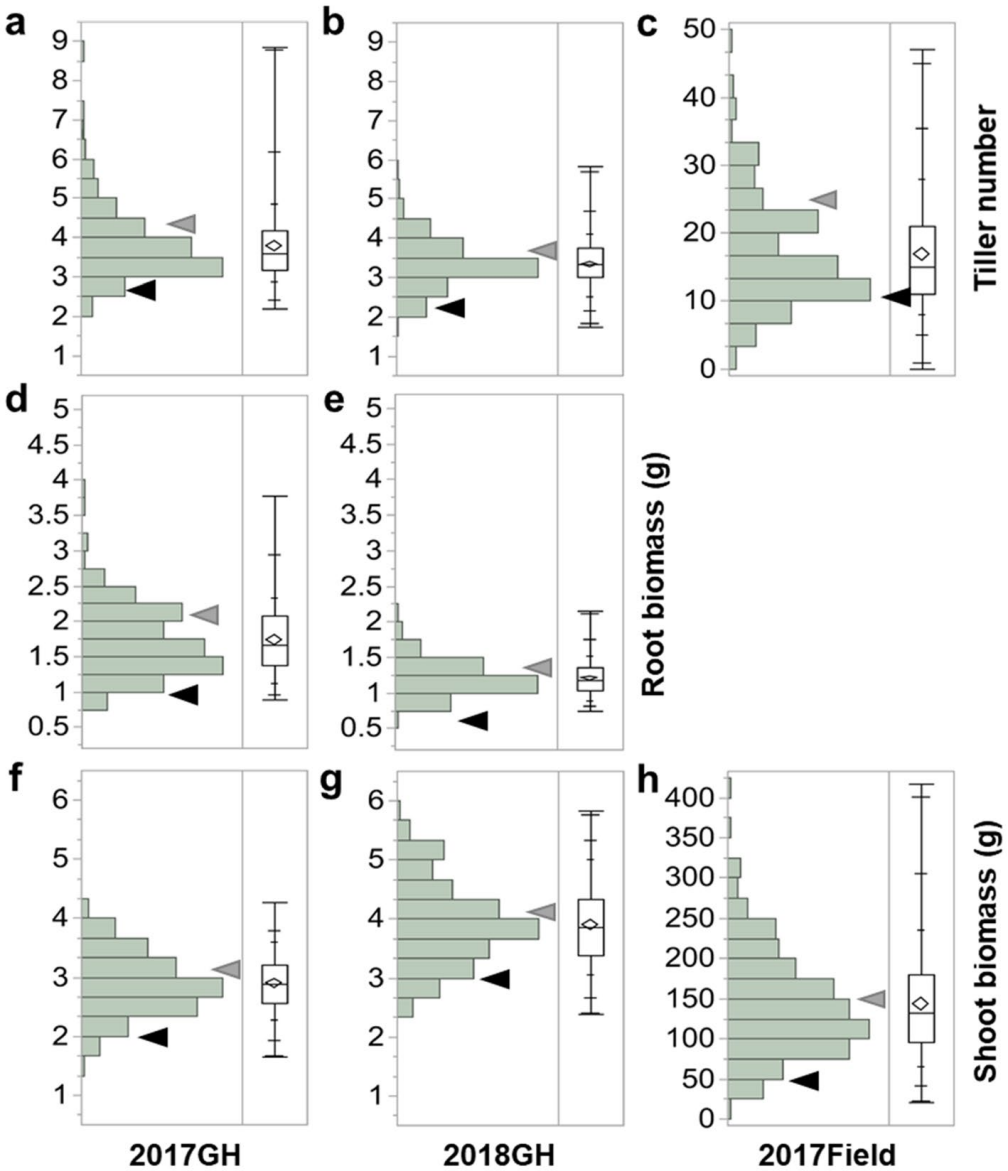
Frequency distribution of traits among the recombinant inbred lines derived from the cross ‘Francis’ x ‘Rondo’ (FR-RILs) plus their parents (Francis black triangles, Rondo gray triangles). The phenotypes for tiller number (**a**–**c**), root biomass (**d**–**e**), and shoot biomass (**f**–**h**) of the 205 and 250 FR-RILs at maximum tillering stage in 2017GH (**a**,**d**,**f**) and 2018GH (**b**,**e**,**g**) greenhouse study, respectively, as well as 250 FR-RILs at harvest stage in the 2017F study (**c**,**h**) are displayed. Distribution of quantile box plot of the traits are shown on the right panel. The LS mean and the 95% confidence interval are represented by a horizontal line and a box, respectively.

The frequency distributions show a wider range in TN and RB among the FR-RILs in 2017GH than 2018 GH (Fig. 1a vs. b, Fig. 1d vs. e, Table S1) while the opposite was seen for the range in SB, with wider phenotypic variance in 2018GH than in 2017GH (Fig. 1f vs. g, Table S1). After division of the FR-RILs into quartile groups per study, TN ranged from 2.2 to 3.2 (1st quartile; Group 1), 3.2–3.6 (2nd quartile; Group 2), 3.6–4.2 (3rd quartile; Group 3) and 4.2–8.8 (4th quartile; Group 4) in the 2017GH while it spanned from 1.8 to 3.0 (Group 1), 3.0–3.3 (Group 2), 3.3–3.8 (Group 3) to 3.8–5.8 (Group 4) in the 2018GH study. The average TN of the Group 4 (4th quartile) was 1.5-fold greater in 2017GH than 2018GH while the means and ranges of the Groups 1–3 were similar or about 1.1-fold greater in 2017GH than in 2018GH (Table S1). Root biomass ranged from 0.9 to 1.4 (Group 1), 1.4–1.7 (Group 2), 1.7–2.1 (Group 3) to 2.1–3.8 (Group 4) in the 2017GH, and from 0.7 to 1.0 (Group 1), 1.0–1.2 (Group 2), 1.2–1.4 (Group 3) to 1.4–2.2 (Group 4) in the 2018GH. Root biomass in the 2017GH was 1.3, 1.4, 1.5, to 1.8-fold greater in all four quartiles from than in the 2018GH. Shoot biomass varied from 1.7 to 2.6 (Group 1), 2.6–2.9 (Group 2), 2.9–3.2 (Group 3) to 3.2–4.3 (Group 4) in the 2017 GH, and from 2.4 to 3.4 (Group 1), 3.4–3.9 (Group 2), 3.9–4.3 (Group 3) to 4.3–6.0 (Group 4) in the 2018GH (Table S1). Thus, in contrast to TN and RB, SB range was about 1.3 to 1.4-fold greater in 2018GH than in the 2017GH study for all four groups (Table S1). Root biomass was not evaluated in the 2017F study, but in comparing the 2017F TN and SB to those of the two GH studies, TN and SB were five to eightfold and 69–98-fold greater, respectively, at harvest stage in the 2017F study than at maximum tillering stage in the GH studies (Fig. 1c,h).

**Figure 2:**
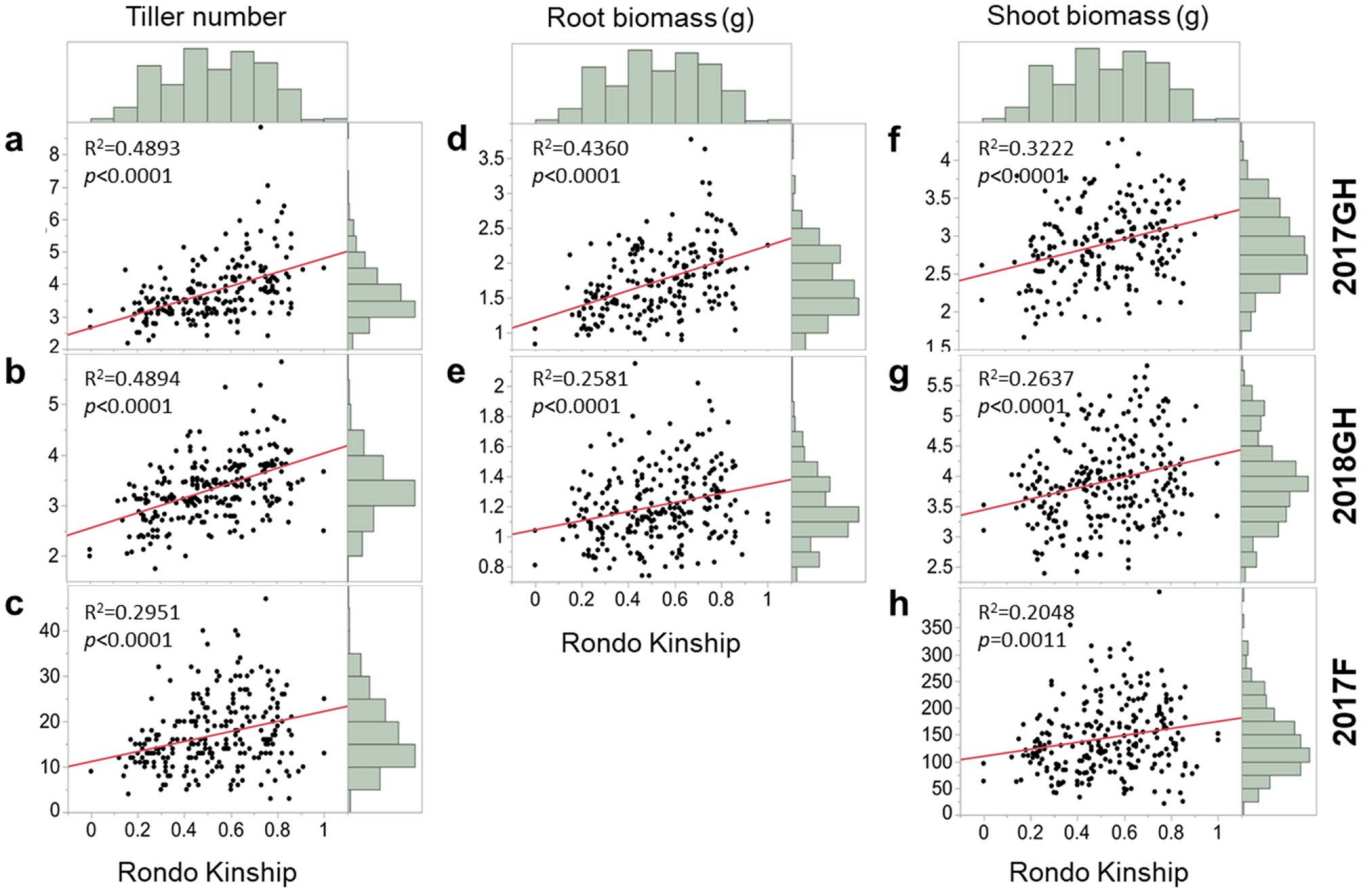
Scatterplot with Pearson’s correlations of Rondo kinship within the FR-RIL mapping population with the traits, tiller number (**a**–**c**), root biomass (**d**–**e**), and shoot biomass (**f**–**h**) at maximum tillering stage in 2017GH (**a**,**d**,**f**) and 2018GH (**b**,**e**,**g**), and at harvest stage in the 2017F study (**c**,**h**).

We further explored the effect of Rondo kinship (i.e., the percentage of alleles in a RIL derived from Rondo) within the FR-RIL mapping population on TN, RB, and SB (Fig. 2). All three traits were positively correlated with Rondo kinship (*p* < 0.001) regardless of developmental stage and environment, but the positive correlations were, in general, stronger at the maximum tillering stage than at the harvest stage. Root biomass had a strong positive correlation with Rondo kinship in 2017GH (R^2^ = 0.44, *p* < 0.0001), but was less strongly correlated in 2018GH (R^2^ = 0.26, *p* < 0.0001) which was likely related to greater trait variance (wider distributions) in 2017GH (0.9–4.8 g) than the 2018GH study (0.7–2.2 g) (Fig. 1d,e, and Table S1). For shoot biomass, correlation with Rondo kinship also showed positive correlation in all three environments (R^2^ = 0.32, *p* < 0.0001 in 2017GH, R^2^ = 0.26, *p* < 0.0001 in 2018GH, R^2^ = 0.20, *p* < 0.0001 in 2017F), but in general the kinship correlations for SB were weaker than those for TN and RB (Fig. 2).

Pairwise Pearson’s correlation analysis was conducted to evaluate relationships between TN, SB, and RB in and across the three studies (2017GH, 2018GH, and 2017F) (Table 1). In general, TN at maximum tillering stage between the 2017GH and 2018GH studies showed a strong positive correlation (R^2^ = 0.62, *p* < 0.0001), with TN at harvest stage in the field study (2017F) being less well correlated with TN at maximum tillering from either the 2017GH or 2018GH study (R^2^ = 0.34, *p* < 0.0001 with 2017GH, R^2^ = 0.35, *p* < 0.0001 with 2018GH). RB showed a strong positive correlation between 2017 and 2018GH (R^2^ = 0.46, *p* < 0.0001) but was not measured in the field study. SB also showed a strong positive correlation between the two greenhouse studies (R^2^ = 0.46, *p* < 0.0001), but there was no correlation with either 2017GH or 2018GH with 2017F (R^2^ = 0.10, ns in both 2017GH and 2018GH) (Table 1). Pairwise comparisons between traits demonstrated that TN tended to have stronger correlation with RB than with SB at the maximum tillering stage, and these correlations in the 2017GH study were slightly stronger than in the 2018GH study.

**Table 1:** Pearson’s correlation coefficients between the three traits, tiller number (TN), root biomass (RB), and shoot biomass (SB) evaluated at maximum tillering stage (MT) in the 2017GH and 2018GH studies as well as at harvest stage (H) in the 2017F study.

Trait	Environ	Stage	Tiller number (TN)			Root biomass (RB)		Shoot biomass (SB)		
			2017GH	2018GH	2017F	2017GH	2018GH	2017GH	2018GH	2017F
			MT	MT	H	MT	MT	MT	MT	H
TN	2017GH	MT	1.00	0.62***	0.34***	0.52***	0.22**	0.37***	0.08^ns^	0.10^ns^
	2018GH	MT		1.00	0.35***	0.35***	0.44***	0.21**	0.35***	0.13*
	2017F	H			1.00	0.18**	0.17**	0.08^ns^	0.05^ns^	0.84***
RB	2017GH	MT				1.00	0.46***	0.53***	0.23***	0.16*
	2018GH	MT					1.00	0.17*	0.45***	0.27***
SB	2017GH	MT						1.00	0.40***	0.10^ns^
	2018GH	MT							1.00	0.10^ns^
	2017F	H								1.00

*p < 0.05, **p < 0.01, ***p < 0.001.

### QTLs associated with TN, RB, and SB across different developmental stages and environments

We used a 7K-rice SNP array (C7AIR) data on 250 FR-RILs and the phenotypic trait LS means from the 2017GH and 2018GH studies, and values from 2017F study to perform linkage mapping and QTL analysis. Results revealed a total of eleven QTLs associated with TN, RB, or SB in one or more of the three studies (2017GH, 2018GH and 2017F) that were identified on rice chromosomes, 1, 3, 4, and 5 (Table 2). Six QTLs associated with TN had LOD scores ranging from 3.00 to 4.69, and one QTL (*qTN3-1*) was identified in more than one environment (2018GH and 2017F). Two RB QTL were identified (*qRB3* and *qRB5*), with one (*qRB3*) being identified in both 2017GH and 2018GH with a notably high LOD (17.02) in 2018GH. Three SB QTL were identified with LOD scores ranging from 3.11 to 3.37. The peaks for *qTN3-1* and *qRB3* were only 213 kb apart on chromosome 3, but this is greater than the threshold of 150 kb used in this study for declaring QTLs as co-located. The additive effects for all QTLs were positive (Table 2) indicating that the Rondo allele increased the trait for all of the TN, RB, and SB QTLs identified.

**Table 2:** QTLs identified by interval mapping for tiller number (TN), root biomass (RB) and shoot biomass (SB) at maximum tillering stage (MT) in the 2017GH and 2018GH studies as well as at harvest stage (H) in the 2017F study (2017F).

Trait	Environment	Stage	QTL	Chr	PeakPos	Start	End	cM	LOD	Effect
TN	2018GH	MT	*qTN1-1*	1	375,814	306,611	1,643,235	0.20	4.42	0.32
	2018GH	MT	*qTN1-2*	1	7,116,232	6,200,385	7,421,797	65.60	4.69	0.30
	2018GH	MT	*qTN3-1*	3	1,469,412	853,802	2,885,423	6.69	3.90	0.31
	2017F	H	*qTN3-1*	3	1,469,412	1,476,335	1,483,361	6.69	4.05	4.5
	2017F	H	*qTN3-2*	3	13,220,538	13,218,051	13,222,231	89.85	3.68	4.5
	2017GH	MT	*qTN3-3*	3	16,409,405	851,612	30,119,566	114.66	3.00	0.45
	2018GH	MT	*qTN4*	4	31,509,863	28,028,583	33,663,600	175.27	3.93	0.28
RB	2017GH	MT	*qRB3*	3	1,256,423	996,213	1,292,063	4.81	6.31	0.37
	2018GH	MT	*qRB3*	3	1,256,423	1,008,584	1,292,063	4.81	17.02	0.27
	2018GH	MT	*qRB5*	5	23,551,364	20,430,655	25,085,545	124.21	3.41	0.12
SB	2017GH	MT	*qSB4-1*	4	23,962,137	6,478,580	26,168,663	109.99	3.29	0.29
	2017GH	MT	*qSB4-2*	4	29,776,057	28,112,993	33,448,911	144.61	3.37	0.28
	2018GH	MT	*qSB5*	5	21,509,332	19,401,404	23,889,378	102.35	3.11	0.38

To further confirm the TN QTLs, SNP allele data were used to classify the FR-RILs into groups of RILs containing 0, 1, 2, 3, 4, 5, or 6 TN alleles. Group means were then evaluated, revealing that increasing the number of Rondo alleles at TN QTLs did increase group mean TN in all three environments (Fig. S1). Similarly, the FR-RILs containing Rondo alleles at the two RB QTLs had higher RB in both 2017GH and 2018GH. However, this pattern was not consistent with the number of Rondo alleles for the three identified SB QTLs in either the 2017GH or 2018GH studies (Fig. S1f,g).

### Evaluation of QTL impacts across different developmental stages and environments

**Figure 3:**
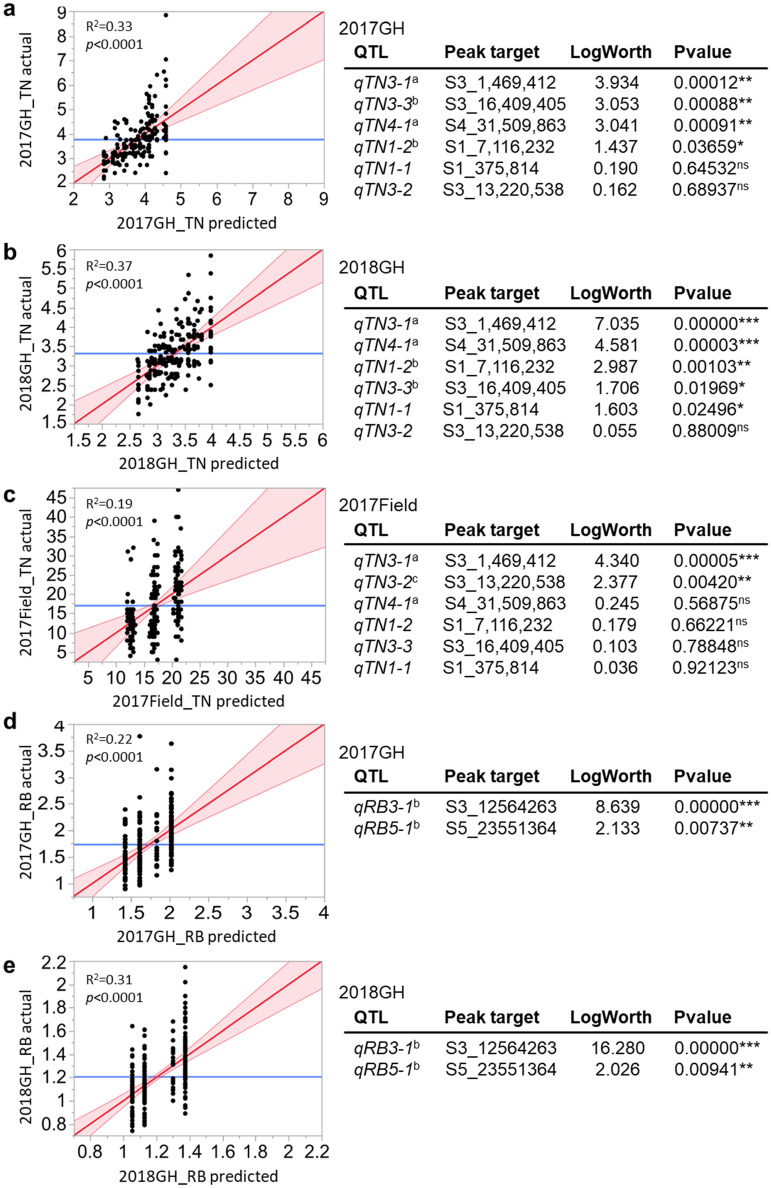
Linear regression of tiller number (TN; **a**–**c**) and root biomass (RB; **d,e**) based on the contribution of the Rondo allele for any of the QTLs identified for TN and RB, respectively, from the 2017GH (**a,d**), 2018GH (**b**,**e**), or 2017F study (**c**). The QTLs that are significantly associated with TN or RB (*p* < 0.05) regardless of environments or developmental stage are marked as ‘a’ while the QTLs that are significantly associated with TN under only one developmental stage, i.e. maximum tillering stage only or harvest stage only, are marked as ‘b’ or ‘c’, respectively. The multiple-regression model expresses the phenotype of TN and RB (y-axis) as a linear function of Rondo allele at TN-QTLs and RB-QTLs identified (x-axis). The proportion of the total phenotypic variation (y-axis) explained by each QTL (x-axis) was calculated as an R^2^ value, from the regressions of each marker/phenotype combination.

The majority of QTL regions identified by interval mapping were found significant in just one environment or at just one developmental stage. Notable exceptions to this were the identification of *qTN3-1* in both the 2018GH and 2017F, and identification of *qRB3-1* in both the 2017GH and 2018GH. To further examine the phenotypic impact of the identified QTLs across different developmental stages and environments, including those in which they were not found significant via interval mapping, for each RIL we used the allele genotypes at all QTLs identified per trait (6 for TN, 2 for RB) to predict the trait phenotypes for each of the growing conditions (2017GH, 2018GH, 2017F) and then performed linear regression of the actual trait data against the QTL-predicted phenotypes (Fig. 3). The multiple-regression model expresses the phenotype of the traits as a linear function of the Rondo allele at each QTL associated with the traits. The proportion of the total observed phenotypic variation explained by each QTL was thereby calculated as an R^2^ value, from the regressions of each marker/phenotype combination. The regression model indicated that the four TN QTLs that explained the observed phenotypic variance within the 2017GH data were *qTN3-1*, *qTN3-3*, *qTN4-1* (*p* < 0.0001), and *qTN1-2* (*p* < 0.05) (Fig. 3a). This differs from the interval mapping results where only *qTN3-3* was identified from analysis of 2017GH data (Table 2). Similar regression modeling indicated that five TN QTLs impacted 2018GH TN, including *qTN3-3* in addition to the four TN QTLs identified from interval analysis of the same 2018GH data. The two TN QTLs found via interval mapping of the 2017F data (*qTN3-1* and *qTN3-2*, Table 2) were also found significant in the regression model analysis (Fig. 3c). Furthermore, the regression modeling indicated that *qTN3-1* explained the most observed variance in TN across different developmental stages and environments (marked as ‘a’ in Fig. 3a–c), while *qTN1-2, qTN3-3* and *qTN4-1* were robust at the maximum tillering stage in the 2017GH and 2018GH studies (marked as ‘b’ in Fig. 3a,b), but not at the harvest stage in 2017F (Fig. 3a,c). In contrast, *qTN3-2* was only effective at the harvest stage (*p* < 0.01) (marked as ‘c’ in Fig. 3c). Similar regression model analysis of the RB data identified the same two QTLs identified by interval analysis, with both *qRB3-1* and *qRB5-1* found effective at maximum tillering stage in 2017 and 2018 (marked as ‘b’ in Fig. 3d,e), but *qRB3-1* (*p* < 0.0001) was more effective than *qRB5-1* (*p* < 0.01) (Fig. 3d,e). RB was not phenotyped in the field study. Per SB, among QTLs identified, only weak correlation was present in any of three conditions (R^2^ = 0.06, 2017GH; R^2^ = 0.04, 2018GH; R^2^ = 0.13, 2017F) (Fig. S2a–c).

**Figure 4:**
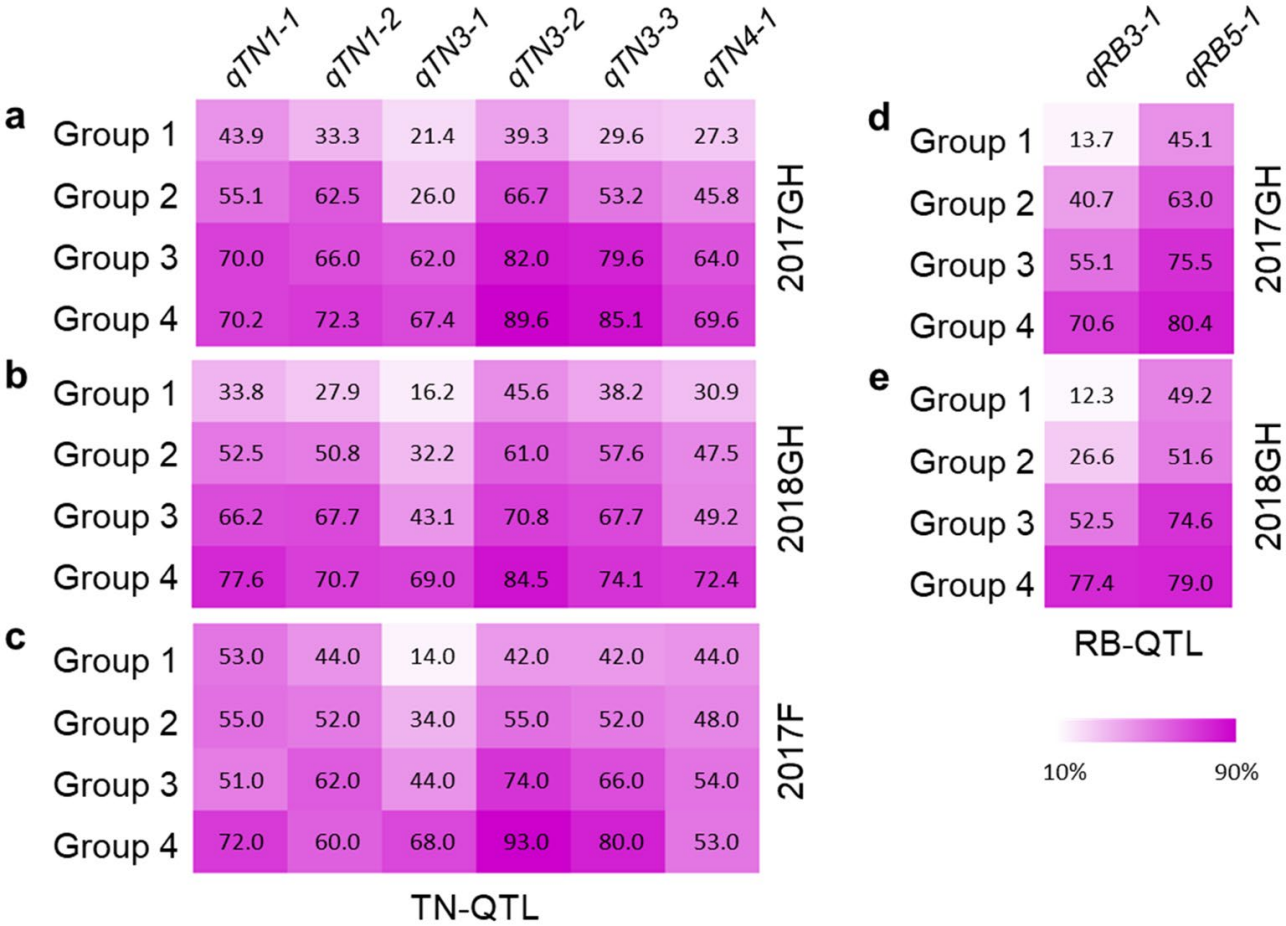
Percentage of lines containing the Rondo allele at each of the identified QTLs that increase tiller number (six QTLs) or root biomass (two QTLs) among four groups of FR-RILs comprising the 1st, 2nd, 3rd, and 4th quartiles based on LS mean tiller number (TN; **a**–**c**) and root biomass (RB; **d**,**e**) per FR-RIL in each experiment (e.g. Group 1 contains the 1/4th of the FR-RILs ranked in the first quartile for having lower TN than the FR-RILs in Group 2, the 2nd quartile, with Group 4 containing the 1/4th of the FR-RILs having the most TN per that study) at maximum tillering stage in the 2017GH (**a**,**d**) and 2018GH (**b**,**e**) study as well as at harvest stage in the 2017F study (**c**).

As another method for examining the effectiveness of each QTL across the three environments, we looked at the four quartile Groups per trait per environment, this time comparing them for the percentage of FR-RILs carrying the Rondo allele for increased TN, RB, or SB at each QTL (Fig. 4). The quartile Groups were independently determined by each growing condition, 2017GH (Fig. 4a,d, Fig. S3a), 2018GH (Fig. 4b,e, Fig. S3b), and 2017F (Fig. 4c) by ranking the measured TN (Fig. 4a–c), RB (Fig. 4d,e), and SB (Fig. S3a–c), and by definition of quartile, the Groups had the same (or very similar) number of FR-RILs per Group. In general, the RILs in the high TN group (the 4th quartile, Group 4) had a higher percentage of Rondo alleles at each of the six TN QTLs compared to Group 3, which generally had more Rondo alleles than Group 2, which had more Rondo alleles than Group 1 for all three growing conditions. The same association between Rondo allele frequency and Group 1 to 4 membership was observed for the two RB QTLs. Sometimes the increase in Rondo allele percentage was incremental across the four groups for all growth conditions, such as for *qTN3-1* or *qRB3-1.* For other QTLs, the difference was more notable between neighboring groups, such as the larger difference in percentage between Group 4 and 3 than between Group 3 and 1 for *qTN1-1* in 2017F (Fig. 4c) and for *qTN4-1* in 2018GH (Fig. 4b).

We performed a chi-square test for each of the 2398 markers to evaluate the potential impact of segregation distortion on detection of QTLs. The chi-squared goodness of fit test showed that 46.5% (1115) of the markers significantly differed from the expected 1:1 Francis to Rondo allele ratio at *p* < 0.05 and 7.6% (182) of the markers had highly significant segregation distortion at *p* < 1e−05. This was not unexpected because segregation distortion is typically observed in *indica* × *japonica* crosses^42^. A published study of reciprocal *indica *×* japonica* crosses found a similar percent of distorted markers (43.7% in one cross and 40.2% in the other at *p* < 0.05) with chromosomes 3, 5, 6 and 12 exhibiting the most severe distortion in regions extending up to a whole chromosome arm^43–45^. At 71.9% of the markers with significant segregation distortion (*p* < 0.05), the Rondo allele was more frequent than the Francis allele, suggesting a bias in distortion toward the *indica* parent. Of the markers representing the peak SNP for each QTL, highly significant (*p* < 1e−05) segregation distortion was noted occurring at the *qTN3-2, qRB5-1, qSB5-1*, (Supp Table 3). Markers at *qTN1-1*, *qTN3-1*, *qTN3-3*, *qRB3-1*, and *qSB4-2* also had significant, but less severe segregation distortion, and *qTN1-2*, *qTN4-1*, *qSB4-1* had no significant distortion. No discernable relationship was observed between the amount of segregation distortion and QTL LOD scores or significance of QTLs in multiple linear regression tests (Table 2, Fig. 3, and Fig. S2). In multiple linear regression, for the QTLs with high segregation distortion, *qTN3-2* was only significant in the field, *qRB5-1* was significant in both greenhouse experiments, and *qSB5-1* was non-significant across greenhouse and field experiments. For the QTLs with moderate segregation distortion, multiple linear regression found *qTN-1-1* to be significant only in the 2018 greenhouse experiment, *qTN3-1* to be significant in both the greenhouse experiments and the field experiment, *qTN3-3* and *qRB3-1* to be significant in both greenhouse experiments, and *qSB4-2* to be nonsignificant across all. Finally, for the QTLs with no evidence of segregation distortion, multiple linear regression found *qTN1-2* and *qTN4-1* to be significant in both greenhouse experiments and *qSB4-1* to be non-significant across all experiments.

At each of the biomass-related QTLs (tiller number, shoot biomass, and root biomass) it was the Rondo allele that was associated with an increase. This was not entirely unsurprising because ‘Rondo’ has substantially high tillering and has high root and shoot biomass compared to ‘Francis’ and thus the predominant Rondo allele may indeed be conferring the high tiller number, and high shoot and root biomass in those regions. However, it is unclear if this consistent allele effect direction in detected QTLs could be partially the consequence of segregation distortion and bias toward Rondo alleles in the RIL population. The segregation distortion concern is somewhat alleviated by examining the QTLs for heading date, a non-biomass related trait. The Rondo allele effects for heading date are a mixture of positive and negative values (Table S2) with two QTLs conferring earlier heading and four conferring later heading and significant segregation distortion was detected at only two of the six heading date QTLs (Table S3). One possibility to explain the excess of segregation distortion at biomass QTLs could be that in the development of the RIL population there was inadvertent selection for high tillering and high biomass, perhaps because these traits impacted plant survival, and this inadvertent selection caused the segregation distortion at QTLs for tillering and biomass.

### Impact of TN-QTLs, RB-QTLs, and SB-QTLs on heading date and yield

**Figure 5:**
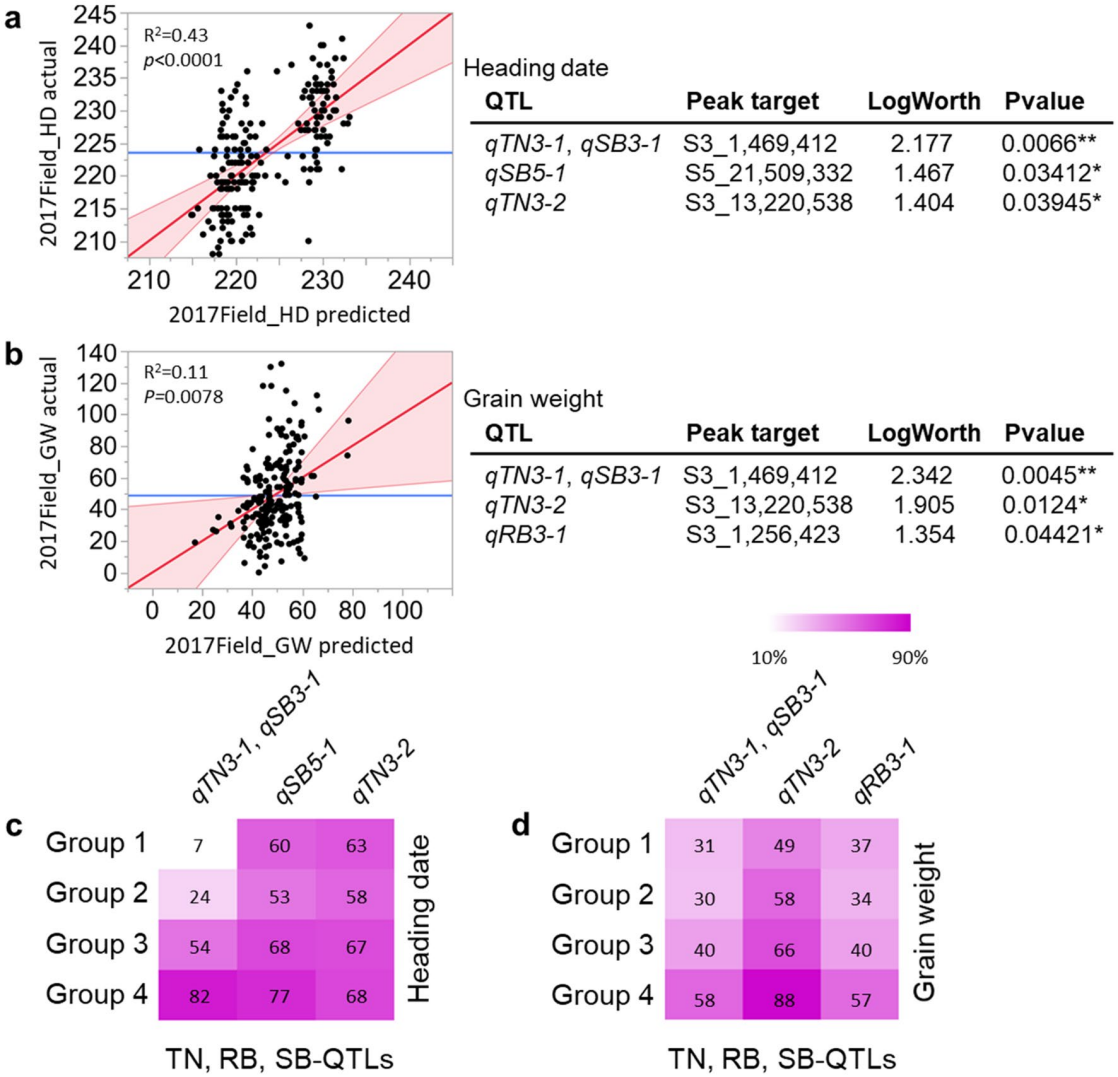
Linear regression of heading date (HD; **a**) and grain weight (GW; **b**) as a function of eleven identified QTLs associated with TN, RB, or SB in one or more of the 2017GH, 2018GH, and 2017F studies. Among the eleven QTLs identified, only the QTLs that are significantly associated with HD and GW (*p* < 0.05) are listed on the right panel. The percentage of RILs containing the Rondo allele at each of the TN-QTLs, RB-QTLs, SB-QTLs in the 1st, 2nd, 3rd, and 4th quartiles, ranked by increasing values for HD (**c**) and for GW (**d**) at the harvest stage in the 2017F are displayed as a heatmap.

To examine the relationship of heading date (HD) and grain weight (GW; yield) with the eleven identified QTLs associated with TN, RB, and SB, as before we calculated the four quartile groups for increasing HD and for increasing GW as observed in the 2017F study and determined the percent of the RIL population containing the Rondo allele at each of the QTLs for TN, RB, and SB. The eleven QTLs associated with TN, RB, and SB showed a stronger positive correlation with HD (R^2^ = 0.43, *p* < 0.0001) than GW (R^2^ = 0.11, *p* = 0.0078) (Fig. 5a,b). Among six TN-QTLs, two RB-QTLs, and three SB-QTLs, three (namely *qTN3-1* (*p* = 0.0066), *qSB5-1* (*p* = 0.034), and *qTN3-2* (*p* = 0.039)) showed significant correlation with HD (Fig. 5a) and three QTLs, *qTN3-1* (*p* = 0.0045), *qTN3-2* (*p* = 0.012), and *qRB3-1* (*p* = 0.04) with GW (Fig. 5b). Interestingly, a clear increase in percent of the RIL population carrying the Rondo allele at the *qTN3-1* was observed to be associated with an increase in HD (Fig. 5c) and an increase in GW (Fig. 5d), i.e. Group 1 to 4, but this was not the case with the other two QTLs, *qSB5-1* and *qTN3-2*. A similar trend was observed for *qTN3-2* and yield rank (49-58-66-88%) (Fig. 5d).

### Candidate genes underlying the TN, RB, and SB QTLs

**Figure 6:**
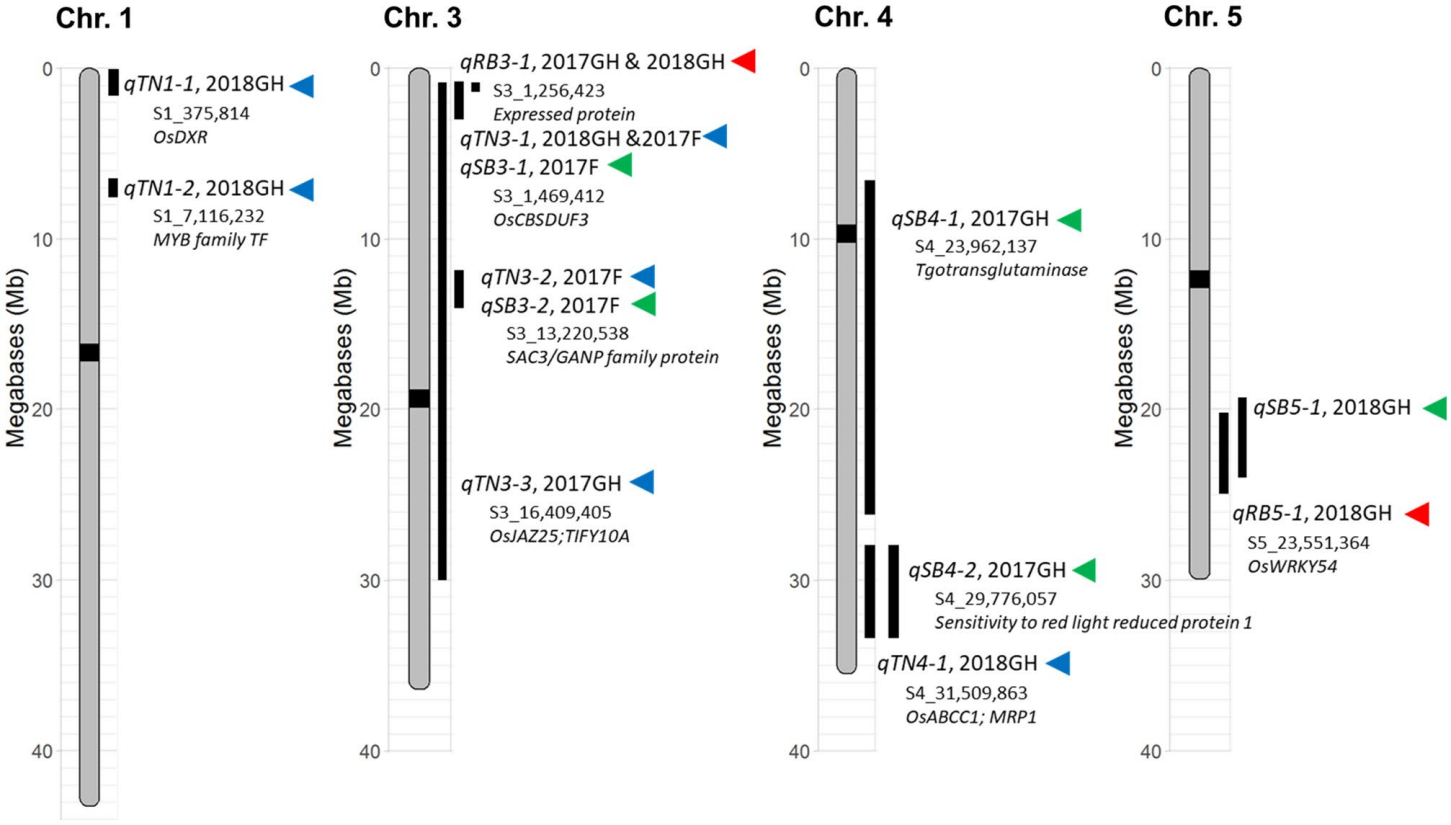
Map of quantitative trait loci (QTL) affecting TN (blue triangle), RB (red triangle), and SB (green triangle) at maximum tillering stage in the 2017GH and 2018GH study as well as at harvest stage in 2017F study. The triangles indicate SNP peak locations. Approximate locations of candidate genes co-localized with the identified TN, RB, and SB QTLs are depicted with italicized gene names.

The TN-QTL found to be most effective at both maximum tillering and the harvest stage (Table 2, Figs. 3 and 6), as well as effective across all three study environments (Figs. 3 and 6) was *qTN3-1.* The annotated gene nearest to the peak SNP (3_1469412) is a Cystathionine β-synthase (CBS) domain containing protein (Os03g03430) which is reported to modulate plant development by maintaining the intracellular redox balance^46,47^. Overexpression of a different CBS domain containing protein (*OsCBSX4*, LOC_Os03g52690) enhanced shoot and root biomass in tobacco plants^48^. Another candidate gene located ~ 150 kb upstream of *qTN3-1* is ***T****illering ****A****nd ****D****warf 1* (*TAD1/TE*; Os3g03150), well-known to be involved in axillary meristem establishment and maintenance. Multiple studies have demonstrated the role of TAD1/TE in regulating the plant-specific shoot branching and tillering, which are major determinants of plant architecture and grain yield^49,50^. Three other TN-QTLs (*qTN3-3, qTN1-2, qTN4-1*) were associated with tiller number at the maximum tillering stage in both greenhouse environments, but not at the harvest stage. The gene nearest the peak SNP for *qTN3-3* is a TIFY family protein, involved in promoting plant growth through jasmonate signaling^51^. For *qTN1-2*, the nearest annotated gene is a MYB transcription factor, and nearest to *qTN4-1* is *OsABCC1* documented to be involved in arsenic (As) detoxification and the As-induced growth inhibition^52^. The *qTN3-2* locus was found effective at the harvest stage but not at the maximum tillering stage. The gene located at the peak of *qTN3-2* is a SAC3/GANP family protein whose role in plants is unknown. However, an important role of the similar SAC3B in plant growth and development has been reported in *Arabidopsis*. *Arabidopsis* SAC3B mutant plants exhibited impaired growth, including shorter primary roots, fewer lateral roots, smaller leaves and shorter inflorescence^53^. The QTL significantly correlated with tiller number only in 2018GH was *qTN1-1*. The gene located at the target region of this QTL is *deoxyxylulose 5-phosphate reductoisomerase* (*DXR*), an early enzyme of the methylerythritol 4-phosphate (MEP) pathway, critical to carotenoid metabolism in rice^54^*.* Strigolactones (SLs), a group of carotenoid-derived plant hormones, are known to impact TN by inhibiting the outgrowth of axillary buds in both monocots and dicots^19,20^.

While interval analysis detected *qRB5-1* in only one GH environment (2018GH), regression model analysis determined it had an effect as well in 2017GH phenotypes. In contrast, *qRB1-1* was identified by both interval and regression analysis in both 2017GH and 2018GH. The peak SNP for *qRB3-1* is located at an expressed protein (Os03g03010) whose function is not yet known. However, ~ 40 kb downstream of *qRB3-1* is *OsMADS50,* whose role in crown root development has been documented. Recent study revealed that *OsMADS50-*overexpressing lines had fewer crown roots, shorter heights, and fewer tillers suggesting OsMAD50 as a negative regulator in root development^55^. The *qRB5-1* peak SNP is located at the *WRKY54* gene. The WRKY transcription factors make up a large, plant-specific gene family with 102 members identified in rice. Extensive studies have revealed that WRKY transcription factors not only play important roles in plant growth and development, but also have functions involved with regulating plant responses to biotic and abiotic stresses. Many of WRKY genes are known to be involved in root development^56^. The genes located at peaks of the three SB-QTLs, *qSB4-1*, *qSB4-2*, and *qSB5-1*, code for transglutaminase (Os04g40300), sensitivity to red light reduced protein 1 (Os04g49930), and a C3HC4 type domain containing zinc finger protein (Os05g36310). However, regression model analysis did not find any of SB QTLs to have an impact on SB (Fig. S2). While no GW-QTL were detected from interval analysis of the 2017F data, when the eleven identified TN-, RB-, and SB-QTLs were included in the linear regression model analysis, the *qTN3-1/qSB3-1*, *qTN3-2*, and *qRB3-1* loci showed significant positive correlation with GW. Linking those identified QTLs with yield needs further confirmation through additional research.

We further identified 6 QTLs associated with heading date (*qHD*) (Table S2). Hydrolase, alpha/beta fold family protein, and retrotransposon protein are in the *qHD3-1* target region, and OsFbox129 and lactose permease-related protein are located in the *qHD3-2* region. Frigida protein is in the *qHD7-1* region, and transposon protein and 3-ketoacyl-CoA synthase 2 are located in the *qHD7-2*. Small auxin-up RNA 31 is in the *qHD8-1* region, and hypothetical protein is located in the *qHD8-2*. Although these measured traits were based only on a single plant, further verification of *qTN* and *qHD* would be needed using larger field plots. No QTL associated with grain weight was identified.

This study identified 11 QTLs affecting TN, RB and SB in an *indica* × tropical *japonica* mapping population at the early vegetative stage as well as at harvest maturity (Fig. 6). The positive additive effects of these QTLs were associated with alleles from the *indica* parent demonstrating the importance of this gene pool as a source of genetic improvement in the predominantly tropical *japonica* germplasm that is used in the USA. Although combining multiple QTLs appeared strongly associated with increasing TN and RB, this is difficult using traditional breeding methods. However, using both interval mapping and multiple regression analysis proved an effective means to select QTLs that are robust across plant growth stages and growing environments with qTN3-1 deemed consistently important. Moreover, the combination of *qTN3-1*, *qTN3-2*, and q*SB5-1*, were demonstrated to have positive relationship with a longer vegetative cycle in the field as evidenced by increased days to heading. Similarly, the combination of *qTN3-1*, *qTN3-2*, and *qRB3-1* were found to be associated with increased grain yield under field conditions. A number of candidate genes were identified in these QTL regions that are linked with plant growth and root development. This knowledge of the QTLs, associated markers, candidate genes, and germplasm resources is of value to rice cultivar improvement programs.

## Materials and methods

### Greenhouse studies (2017GH and 2018GH)

A mapping population comprising 250 F_10_ recombinant inbred lines (FR-RILs) developed from a cross of ‘Francis’ (tropical *japonica*) and ‘Rondo’ (*indica*)^31^ was used in a greenhouse evaluation to study TN, RB, and SB. The study was conducted during 2017 and 2018 in the same greenhouse at the Dale Bumpers National Rice Research Center located in Stuttgart, Arkansas, where temperatures were maintained between 26 and 29 °C and a minimum daylength of 12 h was maintained with supplemental halogen lighting.

Three replications of the FR-RILs and parents were planted in a randomized complete block design, and grown for 6 weeks (maximum tillering stage)^57,58^ from April to May in 2017 and in 2018, following the cultural procedures used in prior TN studies^18,28,31^. Each replication included the parental lines and the 250 FR-RILs, each represented by one plant per pot. Each replication was subdivided among four large tubs 1.2 × 2.5 m in area and 0.3 m deep. Pots (15 cm in diameter, 18 cm tall) were filled to 14-cm depth with sterilized field soil from the Stuttgart, Arkansas site (see below). Three seeds were sown at a depth of 1 cm and pots were kept moist throughout the experiment by maintaining approximately 5 cm water depth in the tub. Starting 1 d after planting, each pot received weekly 50 mL of fertilizer solution (4 g L-1 Jack’s Professional 20–20–20 N–P–K [J.R. Peter], plus 0.17 g L-1 iron chelate [Sequestrene 330 Fe, 10% Fe, Becker Underwood]). At 3 weeks after sowing, seedlings were thinned to one plant per pot. At 6 weeks post planting, water levels were raised to the soil level 3 to 4 days prior to harvesting roots and shoots for each experimental unit.

Tiller number was determined as the total number of stems per plant minus one, to remove the main culm from the stem count. After the stem count, soil was removed from roots by washing in running water. A scalpel was used to divide each plant into shoot and root tissues, separating the roots just below the crown. Tissues were dried at 70 °C for 3 days before being weighed to obtain SB and RB dry weight.

### Field experiment (2017F)

A set of 250 F_10_ FR-RILs, each represented by seed from a single F_9_ plant, was grown at the Dale Bumpers National Rice Research Center field site (Stuttgart, Arkansas) during the summer of 2017. The soil at the site is described as a Dewitt silt loam (fine, smectitic, thermic, Typic Albaqualfs) with a total C content of 0.67% and a total N content of 0.085%. A soil analysis was performed prior to planting followed by application of 44 kg ha^−1^ phosphorous (P_2_0_5_) and 67 kg ha^−1^ potassium (K_2_0). The study consisted of one replication of each of the 250 FR-RILs along with six repeated check plots of the two parents planted as an augmented design. 

The field study was drill-seeded on May 9, 2017 using a Hege 1000 plot planter (Wintersteiger Inc., Salt Lake City, UT). Each 0.46 m^2^ single-row plot was sown with approximately 3 g of seed. All fields were irrigated after planting to assure uniform germination and stand establishment and plots emerged 10 days after planting. Pre- and post- emergence herbicides were applied according to local recommendations to control weeds prior to establishing a permanent flood. On June 15, 2017, 5 weeks after planting, 112 kg ha^−1^ of N as urea was applied and a permanent flood was established and maintained at a depth of 13 ± 5 cm for the remainder of the season. After stand establishment, a single representative plant was isolated in the plot (30 cm) for later harvest. Plots were monitored for days to heading and, at maturity, the total above ground portion of a single representative plant was harvested, the plant dried at 70 °C for 3 days and TN, SB, and total grain weight (GW; yield) were determined.

### QTL analysis

DNA was extracted from leaves of 250 single plants grown in the 2017–2018 winter greenhouse corresponding to the F_10_ generation of FR-RILs harvested in 2017F and evaluated using the 7K-Rice SNP Array (C7AIR) provided by Illumina (San Diego, CA) and processed by Eurofins (Warminster, PA). The SNP calls were manually filtered to remove non-polymorphic SNPs and SNPs with excessively high failure rates or excessive heterozygosity, resulting in a total of 2398 markers after filtering. A straightforward imputation of missing data was implemented using the perl script simple_impute_CSSL.pl that uses a simple rule that a missing data point is imputed to match neighboring markers if found within one megabase on both sides of the missing SNP having matching parental alleles. Heterozygous allele calls were converted to missing data prior to QTL analysis to conform to a RIL population QTL model.

QTL analysis was conducted using the R/qtl2 package. Analyses included a naive model that assumes each RIL is independent and a mixed model that includes a leave-one-chromosome-out (LOCO) kinship matrix. The Log-of-odds (LOD) threshold was established for alpha = 0.05 using 1000 permutations. The locations of significant peaks were determined using the LOD thresholds and 95% Bayesian credible intervals. For interval mapping, we used a minimum LOD score of 3.0. QTLs identified in multiple studies for the same trait were considered to be the same QTL if their peak SNPs were within 150 kb. QTLs were named following the convention of ‘q’ for QTL, followed by an acronym for the trait, followed by a number indicating the chromosome e on which the QTL resides. When more than one QTL for a trait was identified on a chromosome, they are distinguished by adding numbers after dashes, with the added numbers being assigned per their physical order along the chromosome. When considering if QTLs for different traits were co-located, a distance of 150 kb between peak SNPs was again the threshold.

### Statistical analysis

Data analyses were conducted in JMP (version 14.0.0). The least squares (LS) means of each trait for each FR-RIL and parental genotype were calculated across the three replicate plants for each greenhouse study and the one replicate plant for the field study. Frequency distribution of the traits were displayed using a quantile box plot. Pairwise Pearson’s correlation coefficients were calculated between the trait LS means. Because the use of multiple comparisons can increase type I error, appropriately stringent significance thresholds for the r values were determined using Bonferroni’s adjustments^59^. The multiple-regression model was developed by expressing the phenotypic traits as a linear function of the allele states of the QTLs identified for each trait. The proportion of the total phenotypic variation explained by each QTL was calculated as an R^2^ value from the regression of each marker/phenotype combination. The percentage of RILs carrying the Rondo allele at each of the identified QTLs that increase TN, RB, or SB was calculated among the four quartile groups per trait per study, with Group 1 consisting of the 1/4^th^ (25%) of the FR-RILs having the lowest trait values per study (also known as the 1st quartile of the population), Group 2 containing those in the 2nd) quartile, Group 3 being the RILs in the 3rd quartile, and Group 4 consisting of the 25% of FR-RILs with highest trait values, and thus in the upper 1/4th (4th quartile) of the population. Differences in trait means among genotypes (FR-RILs) or among groups of FR-RILs predicted by markers to contain fewer to more numbers of QTL per trait were determined using ANOVA with a *p*-value < 0.05 and using Tukey HSD for multiple mean comparisons using a *p*-value < 0.05. To check for potential segregation distortion, a chi square analysis was performed on all markers using MapDisto v1.7^60^.

### Ethics approval

All methods were performed in accordance with the relevant guidelines/regulations/legislation.

## Supplementary Information

Supplementary Information 1.

Supplementary Information 2.

## Data Availability

The genotype file for ~ 250 mapping population is provided as a supplemental file, and the datasets used and/or analyzed during the current study are available from the corresponding author on request.
