# Supplementary material for: Identification of quantitative trait loci for tillering, root, and shoot biomass at the maximum tillering stage in rice
Source: Sci Rep. 2022 Aug 3;12:13304. doi: 10.1038/s41598-022-17109-y (PMC9349274; doi:10.1038/s41598-022-17109-y)
Supplement: Supplementary file 1 — Supplementary Information 1. [file 41598_2022_17109_MOESM1_ESM.docx]

# Supplementary Material

## Supplemental Figures

**
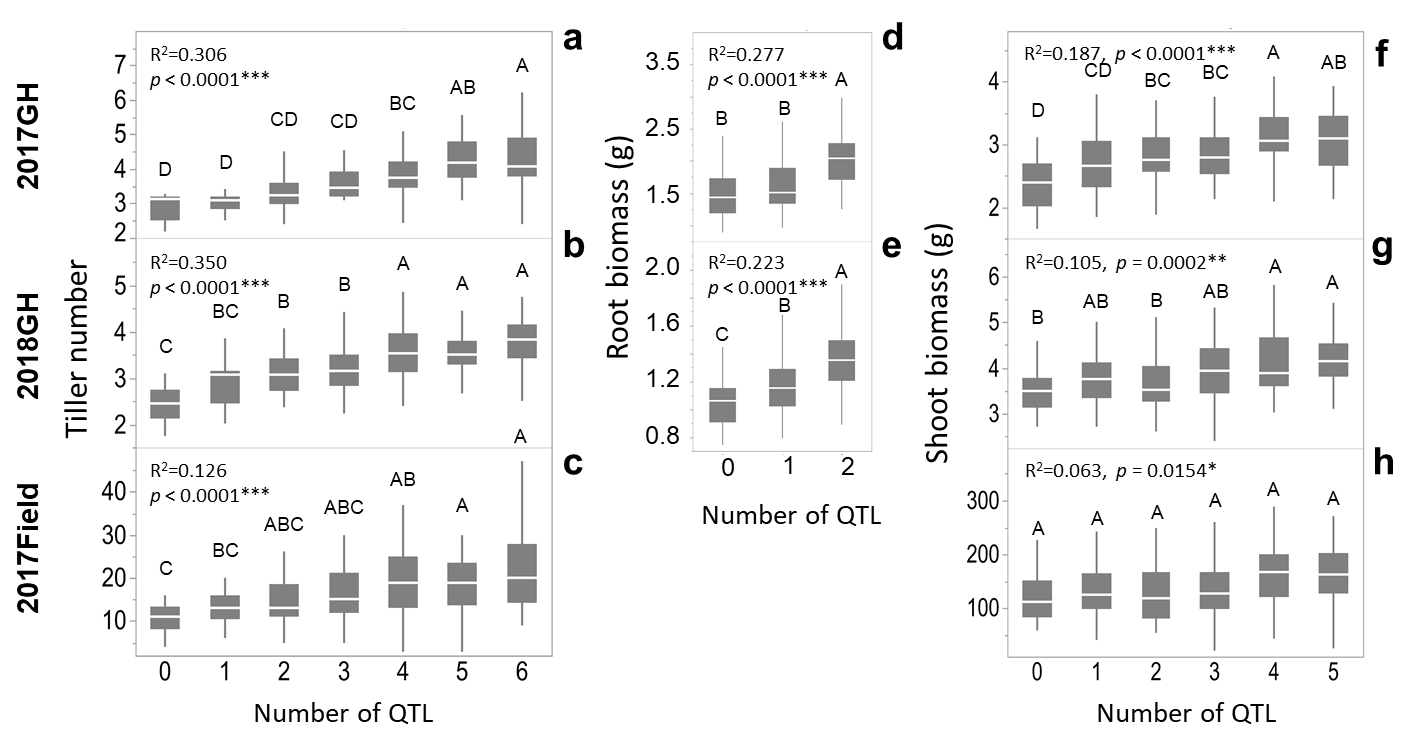
**

**Supplemental Figure 1.** Tiller number (TN; **a**-**c**), root biomass (RB; **d**-**e**), and shoot biomass (SB; **f**-**g**) in the 2017GH (**a**, **d**, **f**), 2018GH (**b**, **e, g**), and 2017F (**c, g**) study by number of QTLs identified for each trait. Letters indicate significant differences within each study for each trait (*p* < 0.05).

**
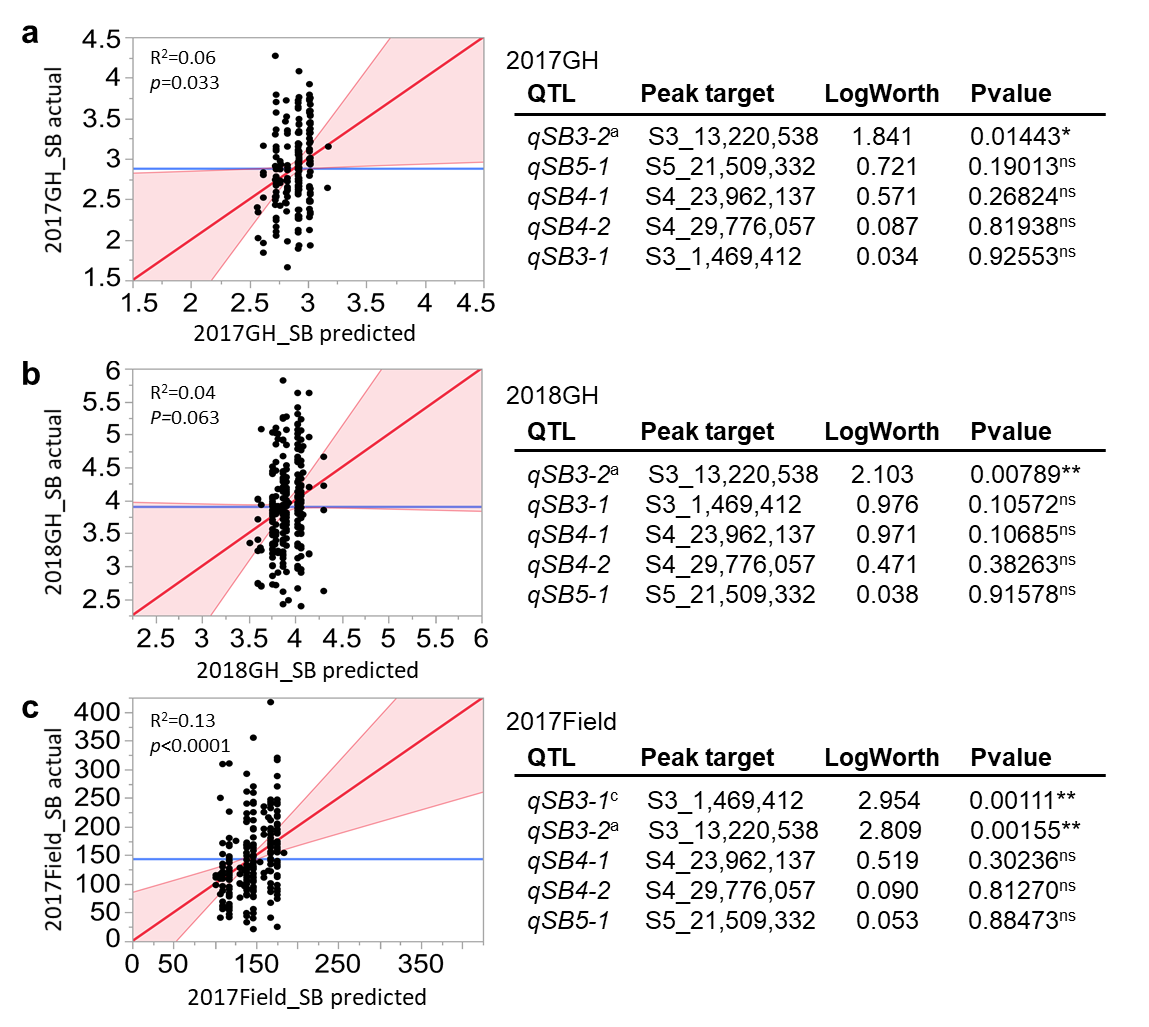
**

**Supplemental Figure 2.** Linear regression of shoot biomass (SB) based on the pattern of genotypic allele, i.e. Francis allele vs Rondo allele, with five identified SB affecting QTLs in 2017GH (**a**) and 2018GH (**b**) study as well as 2017F study (**c**). The QTLs that are significantly associated with SB (*p* < 0.05) regardless of difference in developmental stages (i.e. maximum tillering stage vs harvest stage) and environments (i.e. 2017GH vs 2018GH) are marked as ‘a’ while the QTLs that are only significantly associated with SB under same developmental stage, i.e. maximum tillering stage and harvest stage only, are marked as ‘b’ and ‘c’, respectively. The multiple-regression model expresses the phenotype of SB (y-axis) as a linear function of Rondo allele at SB-QTLs identified (x-axis). The proportion of the total phenotypic variation (y-axis) explained by each QTL (x-axis) was calculated as an R^2^ value, from the regressions of each marker/phenotype combination.


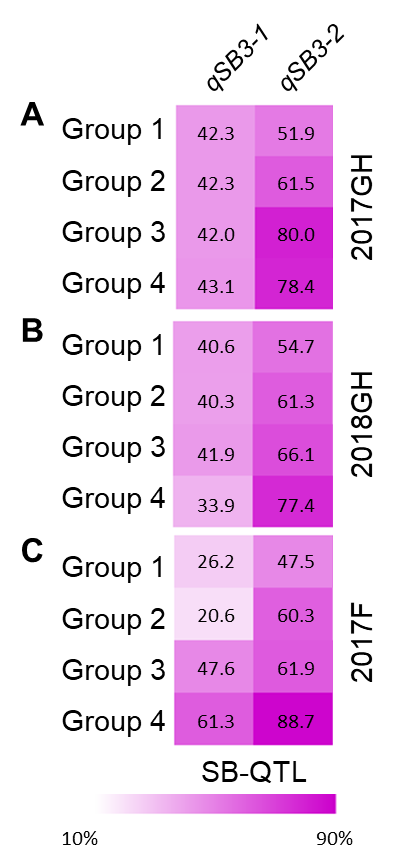


**Supplemental Figure 3.** Percent of lines containing Rondo allele at each of the identified QTLs that increase shoot biomass among four groups, 1^st^, 2^nd^, 3^rd^, and 4^th^ quartile based on the range of shoot biomass (SB) observed in each experiment at maximum tillering stage in the 2017GH (**a**) and 2018GH (**b**) greenhouse study as well as at harvest stage in the 2017F study (**c**).

## Supplemental Tables

**Supplemental Table 1**. LS means of the 25^th^, 50^th^, 75^th^, and 100^th^ quartile group, called Group 1-4, respectively, of the traits. The phenotypes of tiller number (TN), root biomass (RB), shoot biomass (SB), heading date (HD), and grain weight (GW) were evaluated at maximum tillering stage in the 2017 and 2018 greenhouse study (2017GH and 2018GH) as well as at harvest stage in the 2018 field study (2017F). 2017F is the value from one replicate field plant.

Trait Environ. Stage Distribution LSMean

TN 2107GH MT Group 1 1st quartile (0-25%) 2.2-3.2

Group 2 2nd quartile (25-50%) 3.2-3.6

Group 3 3rd quartile (50-75%) 3.6-4.2

Group 4 4th quartile (75-100%) 4.2-8.8

2018GH MT Group 1 1st quartile (0-25%) 1.8-3.0

Group 2 2nd quartile (25-50%) 3.0-3.3

Group 3 3rd quartile (50-75%) 3.3-3.8

Group 4 4th quartile (75-100%) 3.8-5.8

2017F H Group 1 1st quartile (0-25%) 0-11

Group 2 2nd quartile (25-50%) 11-15

Group 3 3rd quartile (50-75%) 15-21

Group 4 4th quartile (75-100%) 21-47

RB 2107GH MT Group 1 1st quartile (0-25%) 0.9-1.4

Group 2 2nd quartile (25-50%) 1.4-1.7

Group 3 3rd quartile (50-75%) 1.7-2.1

Group 4 4th quartile (75-100%) 2.1-3.8

2018GH MT Group 1 1st quartile (0-25%) 0.7-1.0

Group 2 2nd quartile (25-50%) 1.0-1.2

Group 3 3rd quartile (50-75%) 1.2-1.4

Group 4 4th quartile (75-100%) 1.4-2.2

SB 2107GH MT Group 1 1st quartile (0-25%) 1.7-2.6

Group 2 2nd quartile (25-50%) 2.6-2.9

Group 3 3rd quartile (50-75%) 2.9-3.2

Group 4 4th quartile (75-100%) 3.2-4.3

2018GH MT Group 1 1st quartile (0-25%) 2.4-3.4

Group 2 2nd quartile (25-50%) 3.4-3.9

Group 3 3rd quartile (50-75%) 3.9-4.3

Group 4 4th quartile (75-100%) 4.3-6.0

2017F H Group 1 1st quartile (0-25%) 0-95

Group 2 2nd quartile (25-50%) 95-130

Group 3 3rd quartile (50-75%) 130-180

Group 4 4th quartile (75-100%) 180-417

HD 2017F H Group 1 1st quartile (0-25%) 208-219

Group 2 2nd quartile (25-50%) 219-223

Group 3 3rd quartile (50-75%) 223-230

Group 4 4th quartile (75-100%) 230-243

GW 2017F H Group 1 1st quartile (0-25%) 0-30

Group 2 2nd quartile (25-50%) 30-43

Group 3 3rd quartile (50-75%) 43-60

Group 4 4th quartile (75-100%) 60-132

**Supplemental Table 2**. QTLs identified by interval mapping for heading date (HD) at harvest stage (H) in the 2017F.

| Trait | Environment | Stage | QTL | Chr | PeakPos | Start | End | cM | LOD | Effect |
| --- | --- | --- | --- | --- | --- | --- | --- | --- | --- | --- |
| HD | 2017F | H | *qHD3-1* | 3 | 1,359,713 | 1,008,584 | 1,524,051 | 5.87 | 31.34 | 10.56 |
|  | 2017F | H | *qHD3-2* | 3 | 3,671,198 | 3,484,027 | 3,891,759 | 28.94 | 7.4 | 5.88 |
|  | 2017F | H | *qHD7-1* | 7 | 3,847,550 | 2,679,227 | 4,224,652 | 38.92 | 4.04 | 3.72 |
|  | 2017F | H | *qHD7-2* | 7 | 8,756,417 | 8,498,446 | 9,149,950 | 62.48 | 7.94 | 4.76 |
|  | 2017F | H | *qHD8-1* | 8 | 1,031,679 | NA | 2,944,055 | 16.9 | 5.54 | -4.36 |
|  | 2017F | H | *qHD8-2* | 8 | 4,189,990 | 3,943,520 | 4,603,854 | 63.8 | 13.22 | -6.51 |

**Supplemental Table 3.** Chi-square test of the major QTLs identified in this study using RILs developed via single seed descent. ≥ 0.05 ns; < 0.05 and ≥ 0.01 *; < 0.01 and ≥ 0.001 **; < 0.001 and ≥ 0.0001 ***; < 0.0001 and ≥ 1e-05 ****; < 1e-05 *****

| **QTL** | **Marker** | ***x*^2^_1:1_** | ***p*** |
| --- | --- | --- | --- |
| *qTN1-1* | S1_375814 | 5.87 | * |
| *qTN1-2* | S1_7116232 | 2.36 | ns |
| *qTN3-1* | S3_1469412 | 10.16 | ** |
| *qTN3-2* | S3_13220538 | 26.23 | ***** |
| *qTN3-3* | S3_16409405 | 11.66 | *** |
| *qTN4-1* | S4_31509863 | 0.04 | ns |
| *qRB3-1* | S3_1256423 | 5.87 | * |
| *qRB5-1* | S5_23551364 | 23.34 | ***** |
| *qSB4-1* | S4_23962137 | 1.65 | ns |
| *qSB4-2* | S4_29776057 | 8.94 | ** |
| *qSB5-1* | S5_21509332 | 20.25 | ***** |
| *qHD3-1* | S3_1359713 | 10.62 | ** |
| *qHD3-2* | S3_3671198 | 0 | ns |
| *qHD7-1* | S7_3847550 | 0.70 | ns |
| *qHD7-2* | S7_8756417 | 3.43 | ns |
| *qHD8-1* | S8_1031679 | 0.59 | ns |
| *qHD8-2* | S8_4189990 | 7.17 | ** |

## Supplemental Files

**Supplemental File 1**. Genotype file of 261 FR-RILs using 7K-Rice SNP Array (C7AIR) genotyping chip.
